# Biological Pathways Underlying the Longitudinal Association Between Loneliness and Cognitive Decline

**DOI:** 10.1093/geroni/igab046.917

**Published:** 2021-12-17

**Authors:** Kexin Yu, Ted Ng

**Affiliations:** 1 USC, LA, California, United States; 2 ASU , Phoenix, Arizona, United States

## Abstract

Loneliness has been recognized as a risk factor for cognitive decline (CD) in older adults. However, how loneliness “gets under the skin” to influence CD has been conceptually proposed but rarely empirically examined. The purpose of this study is to investigate whether cardiovascular and kidney biomarkers mediate the longitudinal association between loneliness and CD. We used the cross-lagged panel model (CLPM) to examine the hypothesized relationships with 2006, 2010 and 2014 waves of data from the Health and Retirement Study (HRS). Loneliness is measured with 3-item UCLA loneliness scale. Cognitive health was assessed using the total cognition score. Biomarkers considered including HbA1C, total cholesterol, HDL cholesterol, CRP, and Cystatin C. Among all five biomarkers examined, HbA1c significantly mediated the longitudinal association between loneliness and CD. The other biomarkers examined did not mediate the relationship between loneliness and CD. The study findings show loneliness might affect CD through elevating HbA1C levels.

